# Down-Regulating Sphingolipid Synthesis Increases Yeast Lifespan

**DOI:** 10.1371/journal.pgen.1002493

**Published:** 2012-02-02

**Authors:** Xinhe Huang, Jun Liu, Robert C. Dickson

**Affiliations:** 1 Department of Molecular and Cellular Biochemistry and the Lucille Markey Cancer Center, University of Kentucky College of Medicine, Lexington, Kentucky, United States of America; 2 Key Laboratory of Bio-Resources and Eco-Environment of Ministry of Education, College of Life Science, Sichuan University, Chengdu, China; Stanford University Medical Center, United States of America

## Abstract

Knowledge of the mechanisms for regulating lifespan is advancing rapidly, but lifespan is a complex phenotype and new features are likely to be identified. Here we reveal a novel approach for regulating lifespan. Using a genetic or a pharmacological strategy to lower the rate of sphingolipid synthesis, we show that *Saccharomyces cerevisiae* cells live longer. The longer lifespan is due in part to a reduction in Sch9 protein kinase activity and a consequent reduction in chromosomal mutations and rearrangements and increased stress resistance. Longer lifespan also arises in ways that are independent of Sch9 or caloric restriction, and we speculate on ways that sphingolipids might mediate these aspects of increased lifespan. Sch9 and its mammalian homolog S6 kinase work downstream of the target of rapamycin, TOR1, protein kinase, and play evolutionarily conserved roles in regulating lifespan. Our data establish Sch9 as a focal point for regulating lifespan by integrating nutrient signals from TOR1 with growth and stress signals from sphingolipids. Sphingolipids are found in all eukaryotes and our results suggest that pharmacological down-regulation of one or more sphingolipids may provide a means to reduce age-related diseases and increase lifespan in other eukaryotes.

## Author Summary

Studies with rats in the 1930s showed a surprising increase in lifespan when the diet contained 30%–40% fewer calories than normal. This experiment has been repeated on many organisms and is the gold standard for extending lifespan. While we are beginning to understand how calorie restriction regulates lifespan, the mechanisms are complex and much remains to be learned. In the work presented here, we demonstrate a novel way to increase lifespan in the budding yeast *Saccharomyces cerevisiae*. Our strategy is to lower the rate of sphingolipid synthesis either by reducing the synthesis of the first enzyme in the biosynthesis pathway or by using a drug to reduce enzyme activity. This strategy works in part by lowering the activity of a protein kinase, termed Sch9 in yeast and S6K in mammals, both of which are involved in the processes affected by calorie restriction and that control lifespan. In addition, we find that reducing sphingolipid synthesis increases lifespan in ways that are independent of Sch9 and calorie restriction. Since sphingolipids are found in all eukaryotes, our results suggest that pharmacological down-regulation of one or more sphingolipids may provide a means to reduce age-related diseases and increase lifespan in other eukaryotes.

## Introduction

Most of us dream of a long life and wonder if we can increase our time on earth. Except for caloric restriction (CR), there has been little prospect for increasing lifespan. But recent studies offer hope for developing drugs that increase lifespan by reducing the incidence of age-related diseases that kill most humans. The immunosuppressant drug rapamycin has been shown to reduce age-related diseases and increase lifespan in yeasts, flies and mice [1]–[3] and these results have stimulated searches for new ways to enhance lifespan [4]–[6]. Here we introduce a novel strategy to extend the lifespan of *Saccharomyces cerevisiae* cells that involves reducing the rate of sphingolipid synthesis. This strategy increases lifespan at least partly by reducing the activity of the Sch9 protein kinase, a homolog of mammalian ribosomal S6 kinase, both of which regulate lifespan and function downstream of the target of rapamycin (TOR) protein kinase, a well established and evolutionarily conserved regulator of lifespan [7]–[10].

Sphingolipids are recognized as structural components of eukaryotic membranes and as signaling molecules for regulating cell growth and migration, differentiation, apoptosis, senescence and inflammation [11]–[13]. This wide range of functions arises from the variation in carbon chain length, degree and location of unsaturation and hydroxylation along with other modifications of the long-chain bases (LCBs) and fatty acids that are amide-linked to each other to form ceramides, to which polar groups are attached to form complex sphingolipids [14]. Variation in polar head groups further expands the types of sphingolipids found in nature and implies that many more functions await discovery. Although yeast sphingolipids (Figure S1 in Text S1) lack the complexity of those in mammals, *S. cerevisiae* has, nevertheless, been vital in identifying sphingolipid metabolic genes and in understanding sphingolipid functions [15]–[17].

**Figure 1:**
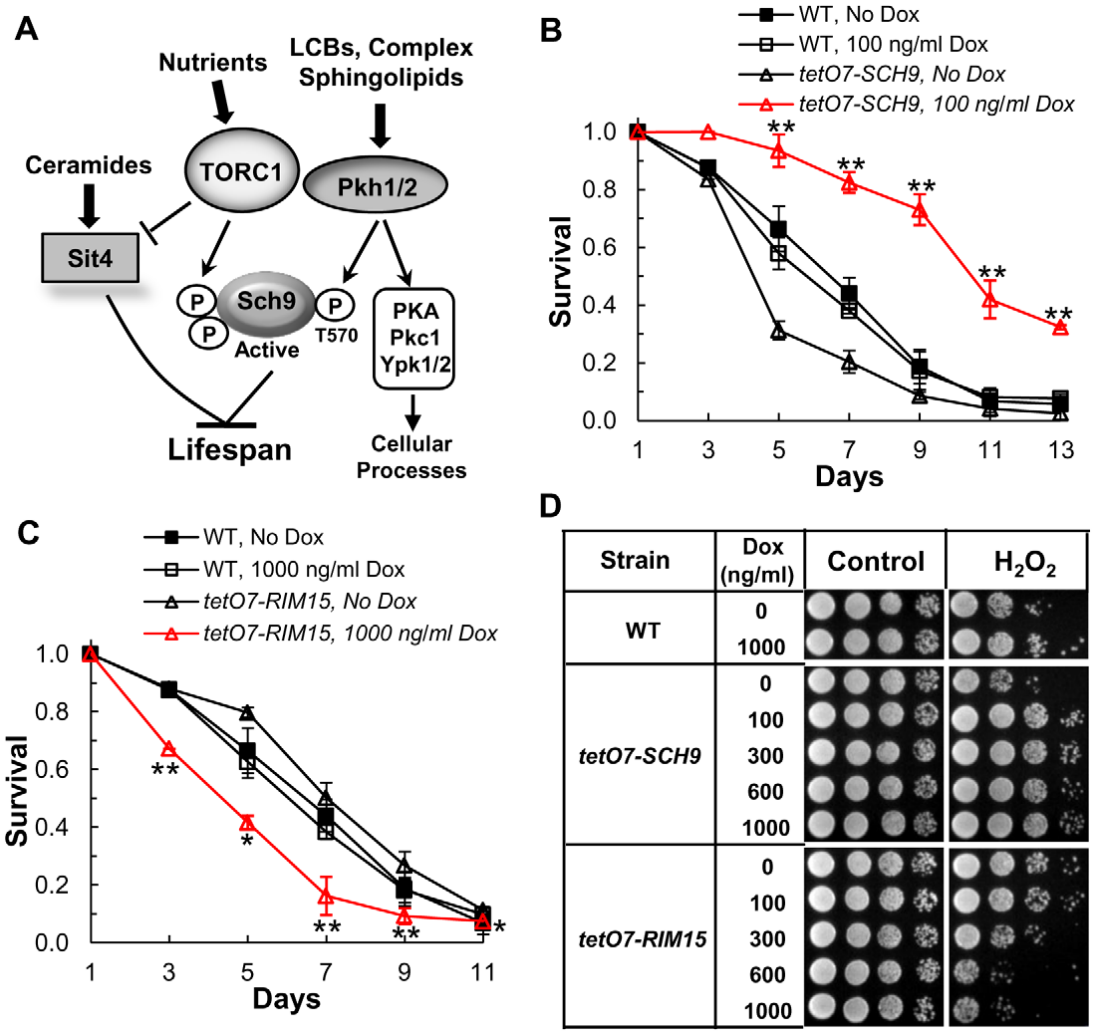
The *tetO7* promoter can be used to regulate CLS. (A) Model showing how sphingolipids are proposed to regulate lifespan. In this model LCBs and perhaps other sphingolipids activate the Pkh1 and Pkh2 protein kinases which phosphorylate residue T570 in the activation loop of the Sch9 protein kinase. Activation of Sch9 also requires phosphorylation of C-terminal residues by the TORC1 kinase. Ceramide-mediated activation of the Sit4 protein phosphatase may also play roles in regulating lifespan as outlined in the Discussion. (B) CLS of *tetO7-SCH9* (RCD952) and WT (R1158) cells +/− Dox treatment. Data show the mean ± SEM of surviving cells (** p<0.01, No Dox vs Dox treatment). Triplicate cultures were used in these and all other CLS experiments. (C) CLS of *tetO7-RIM15* (RCD994) and WT (R1158) cells +/− Dox treatment. Data show the mean ± SEM of surviving cells (* p<0.05, ** p<0.01, No Dox vs Dox treatment). (D) Oxidative stress (H_2_O_2_) resistance of cells from CLS day 1. Strains are: WT, R1158; *tetO7-SCH9*, RCD952; *tetO7-RIM15*, RCD994.

Studies in yeast, worms, flies and mice establish the TOR pathway as a vital regulator of aging and lifespan [1], [2], [8], [10], [18], [19]. Eukaryotes have two types of TOR complexes and TOR complex 1 (TORC1) regulates lifespan. Yeast TORC1 phosphorylates serine and threonine residues in the C-terminus of Sch9 (Figure 1A) [20] while an additional residue, T570, in the activation loop of the kinase domain must be phosphorylated also for Sch9 to be active [20], [21]. Residue T570 is phosphorylated by the redundant Pkh1 and Pkh2 protein kinases, homologs of mammalian phosphoinositide-dependent protein kinase 1 (PDK1) [20]–[22].

Dual regulation of Sch9 provides a way to monitor environmental and cellular cues and integrate them with multiple biological processes necessary for growth and survival. TORC1 senses and is activated by nutrients, particularly amino acids, and growth factors to promote biosynthetic processes such as protein synthesis [10], [23]. In contrast, nutrient depletion or environmental stresses reduce TORC1 activity and biosynthetic processes become less active while catabolic processes, such as autophagy, become more active in order to salvage nutrients and reduce their consumption. Pkh1/2 are also involved in nutrient and stress signaling and activate not only Sch9 (Figure 1A), but also the Ypk1 and Ypk2 protein kinases (homologs of mammalian serum and glucocorticoid-inducible kinase, SGK), Pkc1 and PKA thereby controlling many cellular processes including endocytosis, cell wall integrity, growth, actin dynamics and stress resistance [15], [16], [24], [25].

Sphingolipids are known activators of Pkh1/2 [15], [16], [26], [27] and we posit that they ought to regulate lifespan by controlling the activity of the Pkh1/2-Sch9 pathway. By lowering the rate of sphingolipid biosynthesis, we demonstrate enhancement of chronological lifespan (CLS), a measure of how long non-dividing cells survive in stationary phase. The increase in lifespan is due to Sch9-dependent and independent processes that are also controlled by sphingolipids.

## Results

### Genetic strategy for down-regulating sphingolipid synthesis

In one strategy to determine if sphingolipids regulate CLS we used a tetracycline-repressible promoter cassette (*tetO7*) to regulate gene transcription [28]. The utility of this strategy for studying CLS was verified by using cells having transcription of the *SCH9* gene (*tetO7-SCH9*) or the *RIM15* gene (*tetO7-RIM15*) controlled by the *tetO7* promoter. Based on the roles of Sch9 and Rim15 in lifespan [7], we expected CLS to increase in doxycycline (Dox)-treated *tetO7-SCH9* cells and to decrease in Dox-treated *tetO7-RIM15* cells. We find that a low concentration of Dox (100 ng/ml) significantly increases the CLS of *tetO7-SCH9* cells (Figure 1B) while a higher dose of Dox (1000 ng/ml) is needed to decrease the CLS of *tetO7-RIM15* cells (Figure 1C). In control experiments we find that Dox treatment has no effect on the CLS of the parental strain R1158 (Figure 1B and 1C, WT). Thus, this experimental strategy is able to recapitulate the CLS phenotype of *sch9Δ* and *rim15Δ* mutant cells. It also recapitulates the increased oxidative stress resistance of *sch9Δ* cells and the reduced resistance of *rim15Δ* cells [7] (Figure 1D).

### Down-regulating *tetO7-LCB1* and *tetO7-LCB2* increases CLS

**Figure 2:**
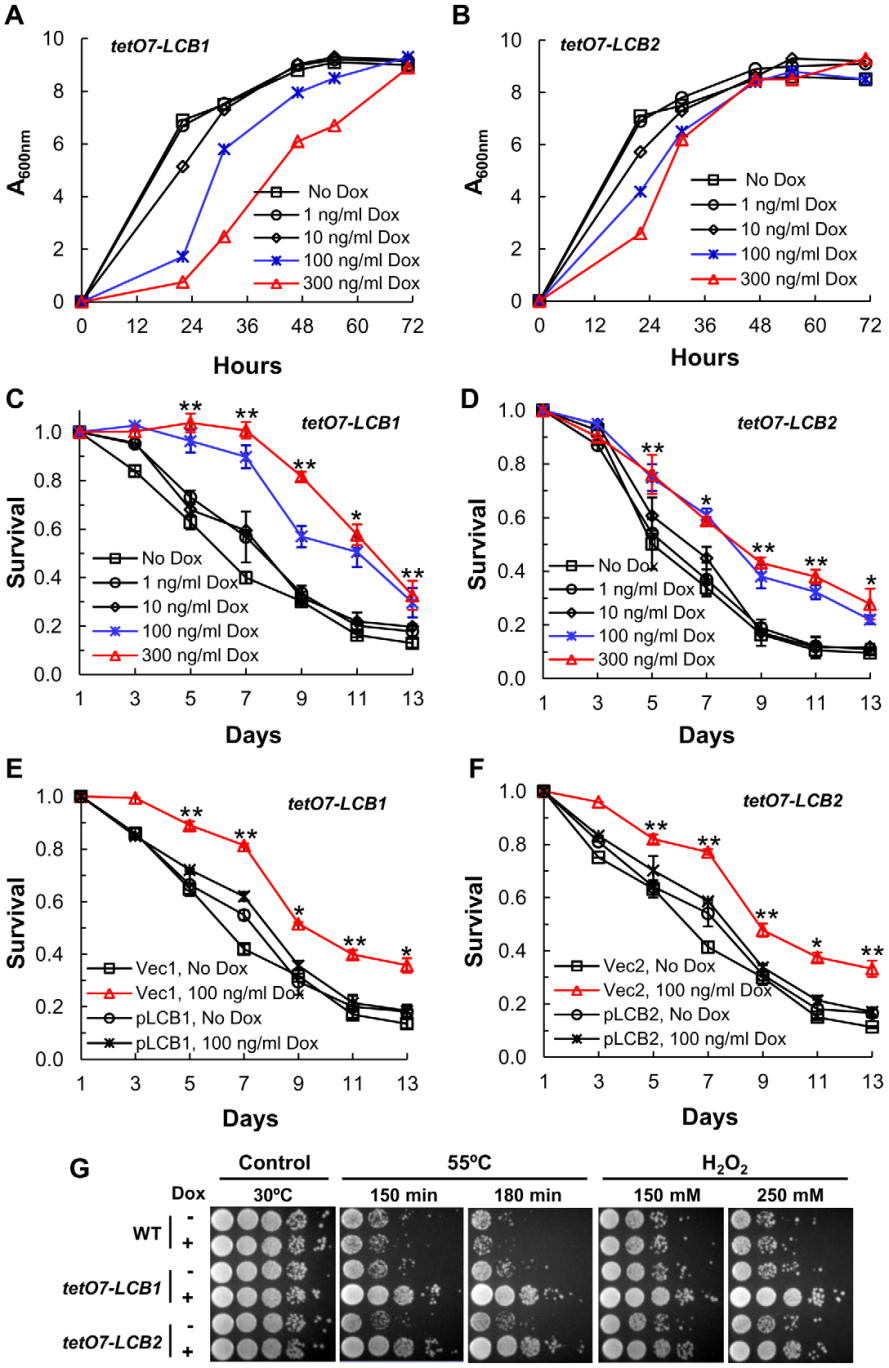
Down-regulating sphingolipid synthesis increases CLS. (A) Growth rate of *tetO7-LCB1* cells (RCD956) treated with Dox to down-regulate expression of the *tetO7* promoter and reduce the rate of sphingolipid synthesis. (B) Growth rate of *tetO7-LCB2* cells (RCD957) +/− Dox treatment. (C) CLS of *tetO7-LCB1* cells +/− Dox treatment. Data show the mean ± SEM of surviving cells (* p<0.05, ** p<0.01, No Dox vs 100 ng/ml Dox). (D) CLS of *tetO7-LCB2* cells +/− Dox treatment. Assays are similar to those presented in (C). (E) Verification that Dox-treatment extends CLS by reducing expression of the *tetO7-LCB1* target gene. CLS of *tetO7-LCB1* cells transformed with an empty vector (Vec1 = pRS315ADE2) or a vector carrying *LCB1* (pLCB1 = pRS315*ADE2-LCB1-5*) is shown. Data represent the mean ± SEM of surviving cells (* p<0.05, ** p<0.01, for Vec1, 100 ng/ml Dox vs pLCB1, 100 ng/ml Dox). (F) Control experiment showing that Dox-treatment extends CLS by reducing expression of the *tetO7-LCB2* gene. CLS of *tetO7-LCB2* cells transformed with a vector carrying *LCB2* (pLCB2 = pRS315-LCB2-B7) or with an empty vector (Vec2 = pRS315) is shown. Data represent the mean ± SEM of surviving cells (* p<0.05, ** p<0.01, for Vec2, 100 ng/ml Dox vs pLCB2, 100 ng/ml Dox). (G) Resistance of Dox-treated WT (R1158), *tetO7-LCB1* (RCD956) and *tetO7-LCB2* (RCD957) cells to heat (55°C) or hydrogen peroxide (H_2_O_2_) stress. Photographs show a ten-fold dilution series of cells (from left to right).

The essential *LCB1* and *LCB2* genes encode subunits of serine palmitoyltransferase (SPT), the first and rate-limiting enzyme in sphingolipid biosynthesis (Figure S1 in Text S1) [15] and we expected that by lowering expression of *LCB1* or *LCB2* it should be possible to lower the level of sphingolipids and extend lifespan via reduction of the sphingolipid-controlled Pkh1/2-Sch9 pathway. We identified concentrations of Dox that reduce the initial rate of growth but not the final cell density of strains with *tetO7-LCB1* or *tetO7-LCB2* alleles, consistent with a lowered rate of sphingolipid synthesis but not low enough to affect the final cell density or mass yield (Figure 2A and 2B). Under these conditions Dox-treated and untreated cells should be in a similar metabolic state upon entering stationary phase and any effect of Dox on lifespan should be due to down-regulation of *LCB1* or *LCB2* rather than to off target effects.

We find that the CLS of *tetO7-LCB1* or *tetO7-LCB2* cells treated with 100 or 300 ng/ml of Dox increases significantly (Figure 2C and 2D). Control experiments demonstrate that this increase in CLS is due to down-regulation of *LCB1* or *LCB2* and not to off target effects of Dox. First, Dox treatment has no effect on the CLS of wild-type R1158 cells, the parent of *tetO7-LCB1* and *tetO7-LCB2* cells (Figure 1B and 1C). Next, we reasoned that introduction of a plasmid-borne wild-type *LCB1* gene into *tetO7-LCB1* cells should preclude Dox from increasing CLS, since the wild-type *LCB1* allele will not respond to Dox and SPT activity should be normal. As expected, the CLS of Dox-treated *tetO7-LCB1* cells transformed with a single-copy vector carrying the *LCB1* gene is reduced compared to cells transformed with just the vector and is similar to cells not treated with Dox (Figure 2E). Analogous experiments with *tetO7-LCB2* cells give similar results (Figure 2F). We conclude from these data that Dox treatment of cells carrying a *tetO7-LCB1* or *tetO7-LCB2* allele increases CLS because expression of the *LCB1* or *LCB2* gene is reduced.

Finally, most experimental manipulations enhance lifespan in part by increasing stress resistance. Thus we anticipated that down-regulating the *tetO7-LCB1* or *tetO7-LCB2* genes by Dox treatment would increase stress resistance and, indeed, resistance to heat and hydrogen peroxide stress did increase (Figure 2G).

### Myriocin treatment increases CLS

**Figure 3:**
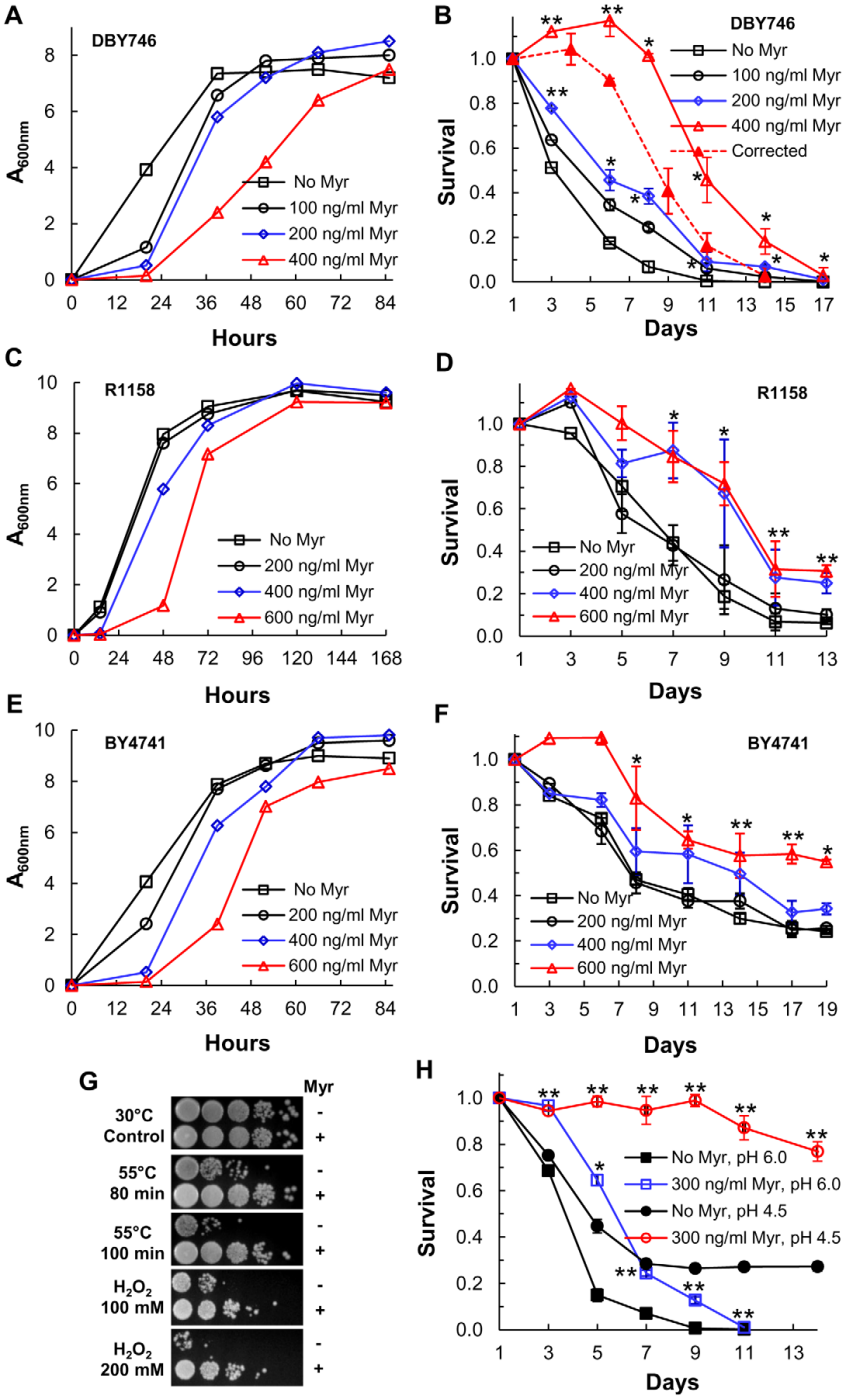
Myriocin treatment extends CLS. (A) Growth rate of DBY746 cells +/− myriocin (Myr) treatment. Myriocin (300 ng/ml) treatment reduces cell size and there are fewer cells/A_600 nm_ in the treated than in the untreated cultures (1.6×10^7^ cells/A_600 nm_ in treated cultures verses 1.8×10^7^ cells/A_600 nm_ in untreated cultures). (B) CLS of DBY746 cells treated as described in (A). Data represent the mean ± SEM of surviving cells (* p<0.05, ** p<0.01, No Myr vs 200 and 400 ng/ml Myr). To compensate for the slow growth and extended growth phase of cells treated with 400 ng/ml of myriocin (solid red line), the viability at the 120 hr time point, CLS day 3, was set as CLS day 1 (dashed red line). (C) Growth rate of R1158 cells +/− myriocin (Myr) treatment. Cells/A_600 nm_ unit are similar to those described in (A). (D) CLS of R1158 cells treated as described in (C). Data represent the mean ± SEM of surviving cells (* p<0.05, ** p<0.01, two-tailed *t*-test for No Myr vs 400 ng/ml Myr). (E) Growth rate of BY4741 cells +/− myriocin (Myr) treatment. (F) CLS of BY4741 cells incubated as described in (E). Data represent the mean ± SEM of surviving cells (* p<0.05, ** p<0.01, No Myr vs 600 ng/ml Myr). (G) Resistance of myriocin-treated DBY746 cells to heat (55°C) or hydrogen peroxide (H_2_O_2_) stress. Photographs show a ten-fold dilution series of cells (from left to right). (H) CLS of DBY746 cells grown in SDC medium buffered to pH 6 or 4.5 +/− myriocin (Myr) treatment. Data represent the mean ± SEM of surviving cells (* p<0.05, ** p<0.01, No Myr vs 300 ng/ml Myr).

A second strategy for determining if sphingolipids regulate CLS uses myriocin, an inhibitor of SPT, to down-regulate enzyme activity. We examined the effect of low doses of myriocin on three strains: DBY746, used extensively to study CLS [7], BY4741, the parent of one of the non-essential yeast gene deletion sets which is often used to study lifespan [8], and a derivative of BY4741, R1158, the parent strain for making *tetO7*-regulated genes [28]. The initial rate of R1158 cell growth is the least impaired by myriocin treatment while growth of DBY746 and BY4741 cells is more impaired (Figure 3A, 3C and 3E). The highest concentration of myriocin used for each strain causes a delay in initial cell growth and increases the time it takes for DBY746 and BY4741 cells to enter stationary phase. However, the final cell density is either the same as or within 20% of the cell density found in the absence of drug, which argues that differences in final cell density or failure to enter stationary phase are not likely to explain the effect of myriocin on CLS. Using these same drug concentrations, there is a robust, dose-dependent and statistically significant increase in the CLS of each strain (Figure 3B, 3D and 3F) with DBY746 cells showing the largest increase (Figure 3B). DBY746 cells treated with 400 ng/ml of myriocin grow slowly and, unlike the other cells, do not stop growing at 72 hrs (CLS day 1), but keep growing up to about the 120 hr time point. This causes an increase in viable cells on CLS days 3 and 6 (Figure 3B, solid red line) and overstates the increase in CLS. To compensate for this extended growth phase, we set the 120 hr time point as CLS day 1 (compared to the 72 hr time point for the other cultures). This corrected survival curve still shows an increase in lifespan (Figure 3B, dashed red line), but not as large an increase as the uncorrected curve (Figure 3B, solid red line).

Myriocin treatment, similar to down-regulation of *LCB1* or *LCB2* expression, increases resistance of DBY746 cells to both heat and hydrogen peroxide stress (Figure 3G). In summary, the data presented in Figure 3 provide additional and independent support for the hypothesis that sphingolipids regulate stress resistance and CLS.

Wild-type yeast cells normally grow to a specific size, but mutation of *SCH9* has the unusual effect of reducing cell size [29]. If a reduction in sphingolipid synthesis increases CLS by reducing Sch9 activity as we predict (Figure 1A), then cell size ought to be reduced. We find that the volume of myriocin-treated DBY746 cells on CLS day 1 is 28% lower than that of untreated cells (Figure S2 in Text S1, 59 verses 82 µm^3^). This reduction is less than the 40% reduction reported for *sch9Δ* cells compared to wild-type DBY746 cells [30], but probably reflects just a partial reduction of Sch9 activity in myriocin-treated cells (see below) compared to no activity in *sch9Δ* cells.

Acetic acid accumulation in the diauxic shift has been proposed as a major cause of cell death, but it can be alleviated by buffering the medium [31]. To eliminate the possibility that myriocin treatment extends CLS by preventing acetic acid killing, we compared the effect of myriocin treatment in buffered and unbuffered medium. Medium was buffered with succinate (200 mM), pH 4.5, because this compound is not metabolized by yeast cells. Myriocin treatment extended CLS in either buffered or unbuffered media (Figure 3H). Thus, myriocin treatment does not extend lifespan simply by increasing resistance to acetic acid and additional support for this conclusion is provided below. Also, we found that the rate of cell death increased because the concentration of iron (1.23 µM) in SDC medium was limiting and we added 3-fold more iron to the medium (indicated in figure legends) to eliminate this variable. The succinate-buffered medium may have a small affect on lifespan due to higher osmolarity as noted before [32], since we found that reducing the concentration from 200 to 50 mM caused a slight reduction in CLS, but no change in pH during the assay. The low pH of this medium is advantageous because the solubility of iron increases greatly at lower pHs [33].

### Down-regulating *LCB1* or SPT lowers sphingolipid levels

If sphingolipids mediate increased CLS, then we predict that down-regulating *tetO7-LCB1* expression by Dox treatment or reducing SPT activity by myriocin treatment should lower sphingolipid levels. We measured LCBs, made primarily as intermediates in the de novo sphingolipid synthesis pathway, as well as the terminal complex sphingolipids found primarily in the plasma membrane. We also measured LCBPs made by phosphorylation of LCBs and thought to primarily represent intermediates in the degradation of LCBs (Figure S1 in Text S1).

**Figure 4:**
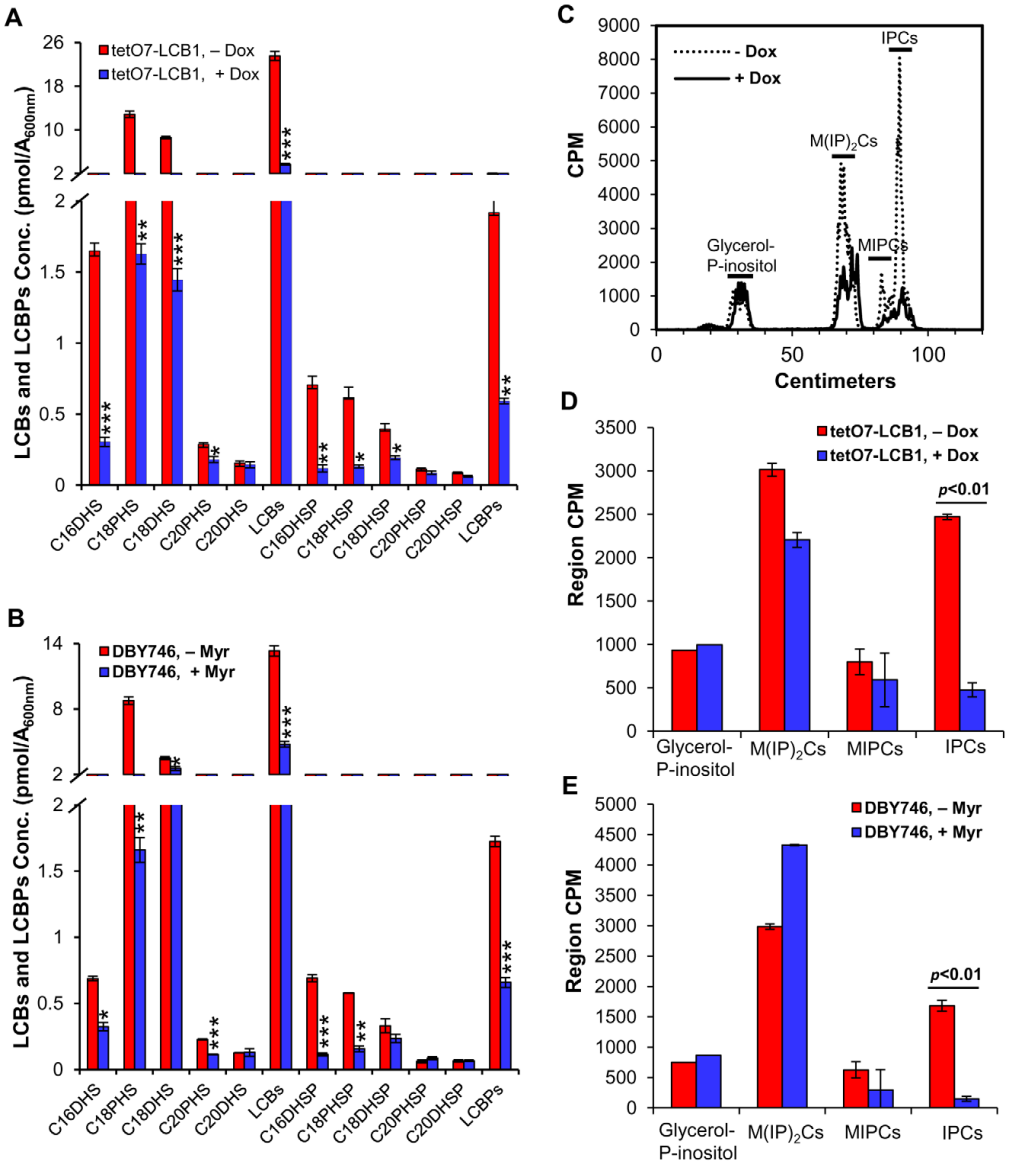
Myriocin or Dox treatment reduces sphingolipid levels. (A) LCBs and LCBPs were quantified in cells grown from an A_600 nm_ of 0.005 to 1 in the presence of absence of Dox (300 ng/ml). Average values for triplicate cultures of *tetO7-LCB1* (RCD956) cells are shown (* p<0.05, ** p<0.01, *** p<0.001, No Dox vs Dox-treated). (B) LCBs and LCBPs were quantified in DBY746 cells treated or not treated with Myr (400 ng/ml) grown the same way as in (A). Average values for triplicate cultures are shown (* p<0.05, ** p<0.01, *** p<0.001, No Myr vs Myr-treated). (C) Complex sphingolipids (IPCs, MIPCs and M(IP)_2_Cs) in untreated and Dox-treated RCD956 cells were grown as in (A) in the presence of [^3^H]-myoinositol and cpm were quantified by scanning the thin-layer chromatogram on a Bioscan apparatus. Glycerol –P-inositol represents the deacylation product of phosphatidylinositol and serves as an internal control. (D) Average cpm +/− SD for [^3^H]-labeled lipids from three cultures analyzed as in (C). (E) Average cpm +/− SD for [^3^H]-labeled lipids from three cultures of DBY746 cells treated or not treated with Myr (400 ng/ml). Cells were grown and analyzed as in (C).

We find that Dox treatment of *tetO7-LCB1* cells, grown the same way as in a CLS assay, reduces total LCBs (C16-, C18- and C20-dihydrosphingosine and C18- and C20-phytosphingosine) by 84% with individual species of LCBs responding uniquely to drug treatment (Figure 4A). The much less abundant LCBPs are also reduced in toto by about 70% (Figure 4A). Dox treatment of wild-type R1158 cells does not produce a statistically significant change in the level of LCBs and LCBPs (data not shown).

Similarly, myriocin treatment of DBY746 cells, grown in the same way as in a CLS assay, reduces total LCBs by 64% and total LCBPs 62% (Figure 4B).

Next, we examined the effect of down-regulating sphingolipid synthesis on complex sphingolipids including inositol-phosphoceramide (IPC), mannose-inositol-phosphoceramide (MIPC) and mannose-(inositol-P)_2_-ceramide (M(IP)_2_C) (Figure S1 in Text S1). There is a statistically significant reduction in IPCs in both Dox treated *tetO7-LCB1* cells and myriocin-treated DBY746 cells (Figure 4C, 4D and 4E). Because MIPCs are not well separated from IPCs (Figure 4C), both types of sphingolipids, not just IPCs, may be reduced in drug-treated cells. There is a tendency for M(IP)_2_Cs to decrease in Dox-treated *tetO7-LCB1* cells but to increase in myriocin-treated DBY746 cells. However, neither change is statistically significant, suggesting that M(IP)_2_Cs are not playing roles in CLS. Our data show that down-regulating the rate of sphingolipid synthesis by impairing activity of the first enzyme in the biosynthesis pathway lowers the concentration of LCBs, LCBPs, IPCs and perhaps MIPCs and some or all of these changes could influence CLS.

### Pkh2 regulates CLS

**Figure 5:**
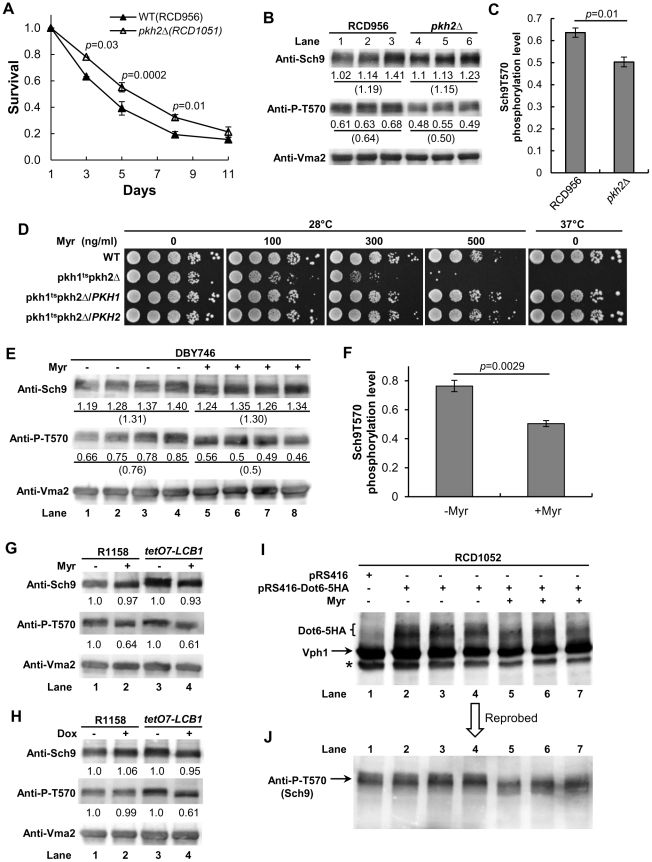
Evidence for the sphingolipid→Pkh1/2→Sch9 pathway. (A) CLS of *pkh2Δ* cells (RCD1051) compared to parental *PKH2* cells (RCD956). Average survival values and statistical significance for eight replicate cultures (total cultures from two different assays) are shown. Statistical significance is indicated for specific time points. These data are nearly identical to published data obtained using a different strain background and culture conditions [34]. (B and C) Immunoblot analysis of Sch9 and P-T570 in the strains used (A) and grown from a starting A_600 nm_ of 0.005 to a value of 1. For each lane the Sch9 signal was normalized to the signal for the Vma2 internal standard (value shown below each lane) and then the P-T570 signal (value shown below the T-570 band) was normalized to this number. Average values are shown in parentheses. (D) Dilution assay (10-fold serial dilution from left to right) for myriocin sensitivity of WT (15Dau/pRS315/pRS316), *pkh1^ts^ pkh2Δ* (INA106-3B/pRS316) and INA106-3B cells transformed with pRS316-PKH1 or pRS316-PKH2. (E) Sch9 and P-T570 were quantified by immunoblotting of extracts made from four cultures of DBY746 cells treated or not treated with myriocin (Myr, 400 ng/ml) while growing from a starting A_600 nm_ of 0.005 to a value of 1. Normalized values were calculated as described in (B). (F) The average of the P-T570 signals from (E) is plotted along with the statistical significance. (G) Sch9 and P-T570 were measured in myriocin-treated or untreated WT (R1158) and *tetO7-*LCB1 (RCD956) cells as described in (B). (H) Sch9 and P-T570 were measured in Dox-treated or untreated WT (R1158) and *tetO7-*LCB1 (RCD956) cells as described in (B). (I) Myriocin treatment reduces Sch9-mediated phosphorylation of Dot6-5HA. RCD1052 cells transformed with the indicated plasmids were grown as described in (B) and immunoblots of extracts were probed with anti-HA antibody or with antibody for the internal standard Vph1 protein. (J) Same blot as shown in panel (I) but reprobed for Sch9 T570 phosphorylation. The asterisk indicates a not specific band.

As further support for our hypothesis that sphingolipids regulate CLS by controlling the Pkh1/2-Sch9 signaling pathway, we sought to show that *PKH1* or *PKH2* or both genes regulate CLS. Recently, deletion of *PKH2* in the diploid BY4743 strain background was shown to produce a small but significant increase in CLS [34]. To verify these data and extend them to a haploid strain background and culture conditions with a different pH and buffering ions, we measured the CLS of a *pkh2Δ* mutant made in the R1158 strain background. We find a small but statistically significant increase in the CLS of *pkh2Δ* cells (Figure 5A). Our data are very similar to results obtained with BY4743 cells where the mean survival interval (area under a survival curve) was 3.10 for wild-type cells compared to 4.05 (34% increase) for *pkh2Δ* cells whereas our values are 4.14 verses 5.23 (29% increase). Together, the published data and our data establish a role for Pkh2 in regulating CLS.

Phosphorylation of residue T570 in Sch9 is critical for protein kinase activity and is mediated in vivo and in vitro by Pkh1/2 [20], [25] and is stimulated by LCBs [21]. If Pkh1/2 regulate Sch9 activity by phosphorylating residue T570, we anticipate less phosphorylation in *pkh2Δ* cells, since this would reflect reduced Sch9 activity and would explain the increase in CLS of *pkh2Δ* cells. We find a statistically significant 20% reduction of T570 phosphorylation in *pkh2Δ* cells compared to wild-type *PKH2* cells which supports the hypothesis that Pkh2 phosphorylates this key Sch9 residue (Figure 5B and 5C).

Another prediction of the proposed signaling pathway going from sphingolipids to Pkh1/2 to Sch9 and other downstream kinases (Figure 1A) is that growth of cells with a low level of Pkh kinase activity ought to be more sensitive to myriocin (reduced SPT activity) compared to cells with a higher level of kinase activity if sphingolipids regulate Pkh activity. Comparison of the myriocin sensitivity of cells with varying combinations of mutant and wild-type *PKH1* and *PKH2* alleles shows that cells with a temperature-sensitive *PKH1* allele or a deletion allele are very sensitive to myriocin at a permissive temperature compared to cells with *PKH1* or *PKH2* or both wild-type genes (Figure 5D). Likewise, deletion of either gene in a *tetO7-LCB1* strain reduces Pkh activity and increases sensitivity to myriocin (Figure S4A in Text S1), moreover, Dox treatment further reduces Pkh activity by lowering sphingolipid levels and impairs growth compared to the parent *tetO7-LCB1* (*PKH1 PKH2*) strain (Figure S4B in Text S1). These data all support the proposed role of sphingolipids as upstream activators of the Pkh1/2 kinases.

### Sphingolipids control phosphorylation of Sch9-T570 and Sch9 substrates

To explore further the hypothesized role of sphingolipids in regulating CLS via the Pkh1/2-Sch9 signaling pathway, we examined phosphorylation of Sch9 residue T570. If sphingolipids control T570 phosphorylation, then reducing SPT activity by myriocin treatment or by Dox treatment of *tetO7-LCB1* cells should decrease phosphorylation. Cells were grown as in a CLS assay, but because drug-treated cells grow slower than untreated cells, comparisons were made with cells grown to similar A_600 nm_ values (∼1) rather than for similar times.

We find a statistically significant 35% reduction in T570 phosphorylation in log phase DBY746 cells treated for 7–8 cell doublings with myriocin compared to untreated cells (Figure 5E and 5F, n = 4 cultures). Also, in parental R1158 (*LCB1*) and *tetO7-LCB1* cells myriocin treatment reduces T570 phosphorylation by 40% (Figure 5G, compare lane 1 with 2 and lane 3 with 4). Lastly, there is a 40% reduction in T570 phosphorylation in Dox-treated *tetO7-LCB1* cells (Figure 5H, compare lanes 3 and 4) but no effect in parental R1158 cells, as expected since *LCB1* is not regulated by the *tetO7* promoter (Figure 5H, compare lanes 1 and 2). These data support the hypothesis that down-regulating sphingolipid levels by genetically or pharmacologically lowering SPT activity reduces T570 phosphorylation and consequently Sch9 activity thereby promoting increased CLS.

In all cases examined (Figure 5E, 5G and 5H), the total level of Sch9 protein is not changed by drug treatment. However, drug treatment does cause a shift of the Sch9 band to a slightly faster migrating form, similar to what is seen when phosphorylation of Sch9 is reduced [20]. In the case where Pkh activity was reduced by deleting *PKH2* rather than by down-regulating sphingolipid synthesis, there was no shift in the mobility of Sch9 to a faster migrating form even though phosphorylation of T570 decreased (Figure 5B). Thus, the band shift we observe suggests that a reduction in sphingolipids triggers dephosphorylation of other Sch9 amino acid residues in addition to T570 and further work will be required to identify the residues involved.

The effect of myriocin treatment on the phosphorylation level of the Sch9 substrate Dot6 was also examined. Sch9 regulates ribosome biogenesis in part by phosphorylating transcriptional repressor proteins including Dot6, Tod6 and Stb3 [35], [36]. Dot6 controls transcription of ribosome biogenesis [37] genes that are essential for ribosome formation. We find that Dot6-5HA in myriocin-treated cells migrates faster on a denaturing gel than it does in untreated cells (Figure 5I), indicating reduced phosphorylation by Sch9 [36]. To directly demonstrate reduced Sch9 activity in these myriocin-treated cells, the blot shown in Figure 5I was reprobed for T570 phosphorylation. As found in other myriocin-treated cells, there was a reduction in T570 phosphorylation and an increase in Sch9 mobility, both indicative of reduced Sch9 activity (Figure 5J). Together, the data shown in Figure 5 demonstrate that down-regulating sphingolipid synthesis by treatment of cells with myriocin or Dox reduces Sch9 activity. Reduced activity is at least partly responsible for the increase in CLS we observe in drug-treated cells (Figure 2, Figure 3, and Figure 7).

### Down-regulating sphingolipid synthesis enhances CLS by Sch9-dependent and -independent mechanisms

**Figure 6:**
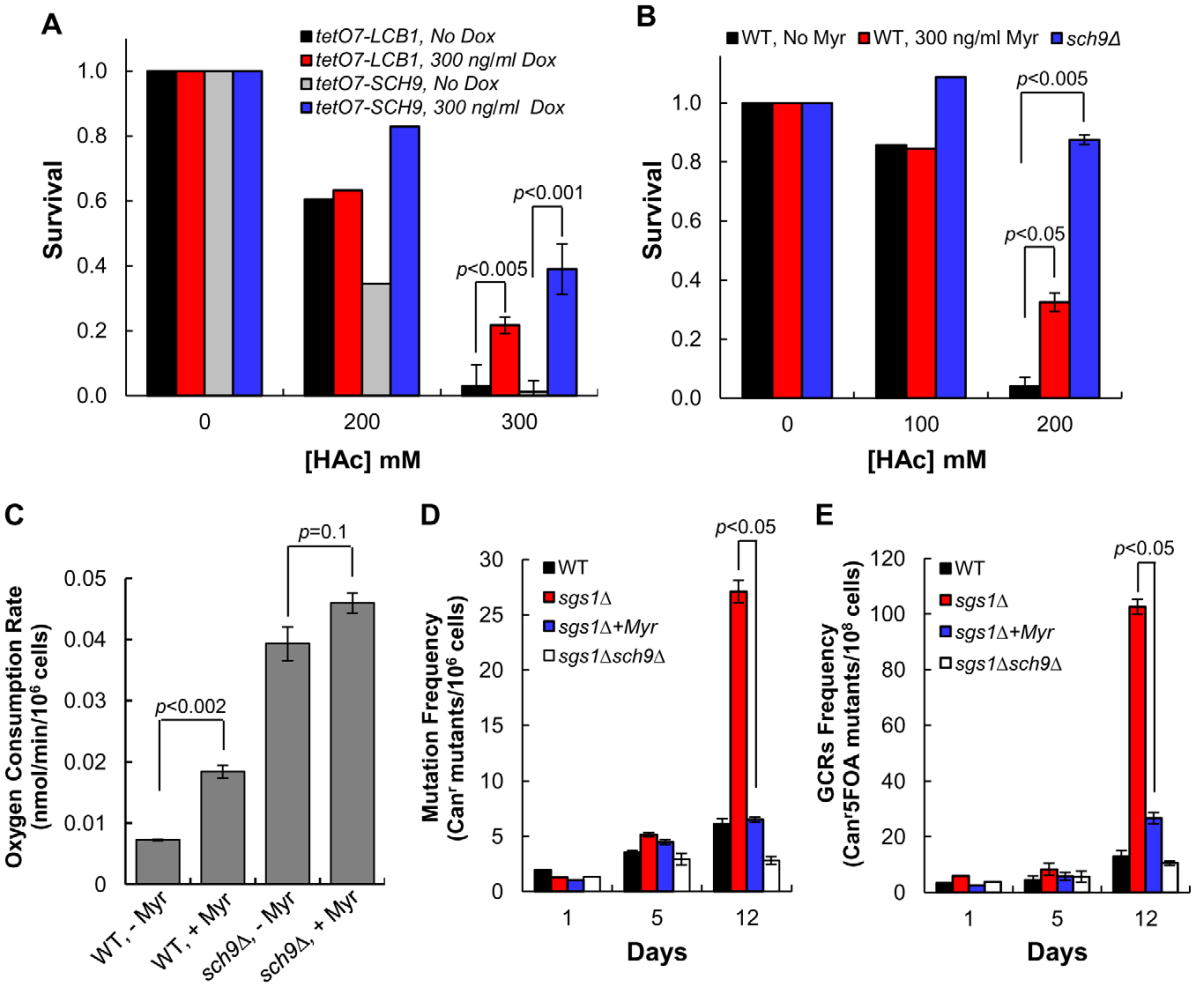
Down-regulating sphingolipid synthesis enhances CLS by Sch9-dependent mechanisms. (A) Doxycycline treatment enhances resistance of *tetO7-LCB1* (RCD956) and *tetO7-SCH9* (RCD952) cells to acetic acid. Cells from CLS day 1, grown in the presence or absence of Dox (300 ng/ml, were treated with acetic acid. (B) Myriocin treatment enhances resistance of DBY746 (WT) cells to acetic acid. Cells were grown and treated with myriocin (Myr) as described in (A). The *sch9Δ* strain was PF102. (C) The rate of oxygen consumption was measured in WT (DBY746) or *sch9Δ* (PF102) cells treated on not treated with myriocin (400 ng/ml). Cells were grown as described in panel B of Figure 5. Because cell volume is affected by myriocin treatment and deletion of *SCH9*, we measured cell volumes and normalized the values to the volume of 10^6^ WT cells grown without myriocin treatment. Average values for four cultures and statistical significance are indicated. (D) Myriocin treatment (300 ng/ml) reduces the frequency of Can^r^ mutations in *sgs1Δ* cells. Cells were grown in SDC medium (pH 4.5, 3× iron). Values represent the average for triplicate cultures started at an A_600 nm_ of 0.05. Strains are: WT, RCD1009; *sgs1Δ*, RCD1010; *sgs1Δ sch9Δ*, RCD1011. (E) Myriocin treatment reduces the frequency of gross chromosomal rearrangements in *sgs1Δ* cells. Strains, culture conditions are the same as described in (D).

Part of the mechanism for increased CLS in *sch9Δ* cells is greater resistance to oxidative, heat and acetic acid stress [7], [31]. If sphingolipids are regulating CLS by Sch9-dependent mechanisms, then reducing the rate of sphingolipid synthesis should increase stress resistance. Indeed, resistance to heat and oxidative stress do increase (Figure 2G and Figure 3G) as does resistance to acetic acid when *tetO7-LCB1* cells are treated with Dox, and resistance is nearly as robust as in *sch9Δ* cells (Figure 6A). Likewise, treating DBY746 cells with myriocin increases resistance to acetic acid albeit resistance is not enhanced as much as in *sch9Δ* cells (Figure 6B). These data support the hypothesis that sphingolipids regulate CLS by Sch9-dependent mechanisms.

Deletion of *SCH9* is known to extend CLS by enhancing respiration [38], [39]. To determine if myriocin treatment also extends CLS by inducing respiration we measured oxygen consumption. We find a more than 2-fold, statistically significant increase in oxygen consumption in myriocin-treated cells compared to untreated cells (Figure 6C). In contrast, there is no statistically significant stimulation of oxygen consumption in *sch9Δ* cells treated with myriocin, indicating that Sch9 mediates most if not all of the effect of myriocin on oxygen consumption (Figure 6C).

Sch9 also affects aging and lifespan by controlling genome stability [40], [41]. For example, *sgs1Δ* cells display premature age-dependent genome instability that is prevented by deletion of *SCH9* [42]. Sgs1 is a member of the RecQ helicase family of DNA unwinding proteins which maintain genome stability. Sgs1 is related to human RecQ helicases WRN, defective in the premature aging disorder Werner's syndrome, and BLM, defective in the cancer-prone disorder Blooms' syndrome [42]. If sphingolipids control Sch9 activity, then age-dependent genome instability in a *sgs1Δ* strain should be prevented by treating cells with myriocin.

Genome instability was quantified by measuring the frequency of Can^r^ mutations. As in previous studies [42], we find Can^r^ mutations accumulating at a faster rate in *sgs1Δ* cells during chronological aging and by day 12 they are 4-fold more abundant than in wild-type cells (Figure 6D). Deletion of *SCH9* reverses the effect of the *sgs1* mutation since cultures of *sgs1Δ sch9Δ* cells have fewer Can^r^ mutants than the wild-type cultures. Notably, treating *sgs1Δ* cells with myriocin reduces the frequency of Can^r^ mutants to the wild-type level at all times during a CLS assay (Figure 6D).

In a second assay of genome instability the frequency of gross chromosomal rearrangements (GCRs) was measured [42]. By day 12 there are five times more GCRs in *sgs1Δ* cells than in wild-type or *sgs1Δ sch9Δ* cells (Figure 6E). Again, myriocin treatment reduces the frequency of GCRs about 75% at day 12 in *sgs1Δ* cells, very similar to the reduction obtained by deleting *SCH9* (*sgs1Δ sch9Δ* cells, Figure 6E). In summary, the data presented in Figure 6 support the hypothesis that sphingolipids regulate CLS by Sch9-dependent mechanisms.

**Figure 7:**
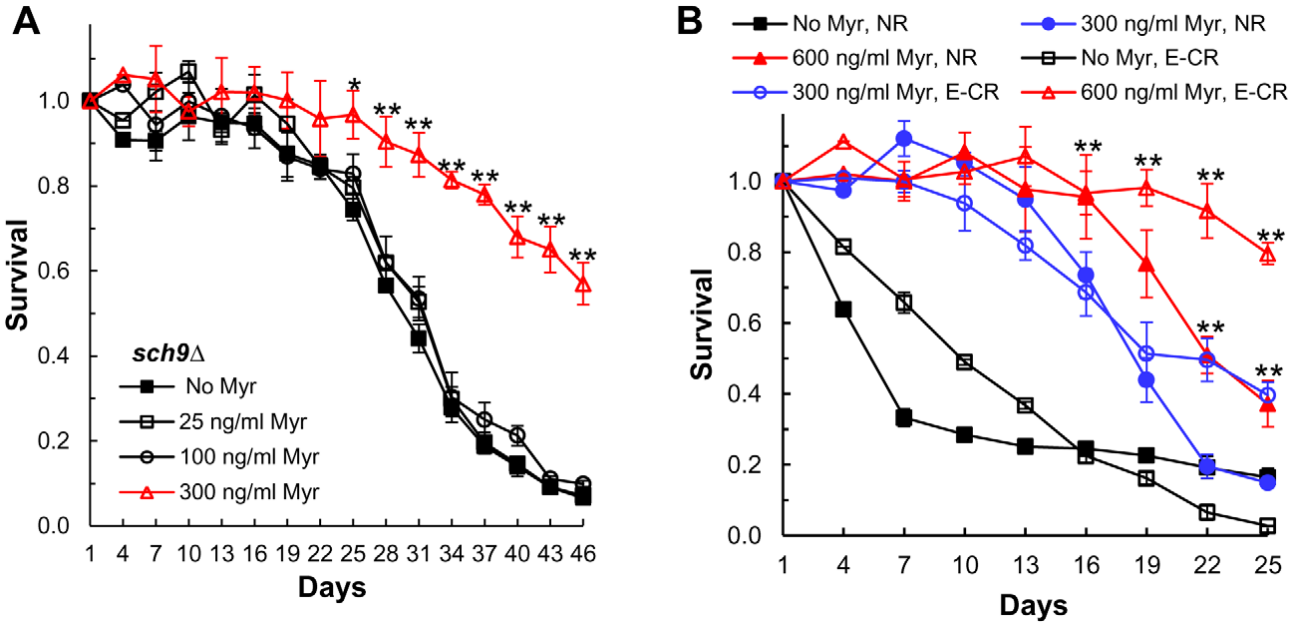
Myriocin enhances CLS by mechanisms that are independent of Sch9 and caloric restriction. (A) CLS of *sch9Δ* cells (PF102) +/− Myr treatment. Data represent the mean ± SEM of survival (* p<0.05, ** p<0.01, No Myr vs 300 ng/ml Myr). (B) CLS of WT (DBY746) cells grown in SDC medium (2% glucose, pH 4.5, 3× iron) +/− Myr for 3 days was measured. After 72 hrs of incubation (CLS day 1) the medium in half of the cultures was switched to water (E-CR) while the other half were not switched to CR (NR). Water was replaced every third day with fresh water to remove nutrients released from dead cells. Data represent the mean ± SEM of viable cells in triplicate cultures (** p<0.01, No Myr vs 300 or 600 ng/ml Myr, E-CR cultures).

Finally, if myriocin enhances CLS entirely by mechanisms requiring Sch9, then myriocin should not enhance CLS in *sch9Δ* cells. We find that a dose of 25 or 100 ng/ml myriocin has no effect on the CLS of *sch9Δ* cells, but a higher dose of 300 ng/ml produces a statistically significant increase in CLS starting around day 22 (Figure 7A). Thus, myriocin most likely extends CLS in the early and middle time-frame of a CLS assay by Sch9-dependent mechanisms, but at later times the enhancement in lifespan is independent of Sch9. Possible mechanisms for the Sch9-independent effects on CLS are presented in the Discussion.

### Down-regulating sphingolipid synthesis extends CLS in caloric-restricted cells

Since calorie restriction increases the CLS of *sch9Δ* cells [30], we determined if myriocin produces an effect similar to CR. CR was implemented by switching cells on day 3 from culture medium to water. This extreme form of CR was used because it prevents adaptive regrowth and eliminates regrowth as an explanation for myriocin-induced CLS extension [40]. We find that myriocin produces a dose-dependent increase in the CLS of cells subjected to extreme CR (Figure 7B). Thus, myriocin extends CLS in *sch9Δ* cells similar to CR treatment, extends CLS even under conditions where adaptive regrowth does not occur, and programs cells for an extended lifespan prior to the end of the diauxic shift (prior to shifting cells to water). In addition, these data further support the conclusion that myriocin does not simply extend CLS by protecting cells from acetic acid killing, since replacing the culture medium with water and changing the water every third day eliminates acetic acid accumulation. Finally, we find that myriocin also enhances CLS when CR is applied by reducing the concentration of glucose from 2 to 0.5% (Figure S3 in Text S1).

## Discussion

The desire for a long and healthy life is universal, but most of us will succumb sooner than hoped for due to age-related disorders, particularly cancer, cardiovascular disease and diabetes. Fortunately, strategies that enhance lifespan in model organisms work to a large degree by delaying the onset of these diseases. A striking example of this relationship is seen in monkeys during CR where the incidence of age-related diseases decreases while lifespan increases [43], [44]. CR is not a practical strategy to extend lifespan in humans, but pharmacological agents are, and the rapamycin-type compounds seem quite promising [4]–[6]. As promising as rapamycin and related drugs are for improving human health and extending lifespan, they may have unforeseen drawbacks and be suitable only for individuals with specific genotypes. Hence, other strategies are needed.

### Sphingolipids regulate yeast lifespan

Our data show that down-regulating sphingolipid synthesis produces an increase in the CLS of *S. cerevisiae* cells (Figure 2, Figure 3, and Figure 7) and that part of this increase depends on the Sch9 protein kinase (Figure S2 in Text S1, Figure 5, Figure 6, and Figure 7A), an established regulator of lifespan. We show that CLS can be increased by lowering sphingolipid synthesis via manipulation of SPT, the initial enzyme in sphingolipid biosynthesis (Figure 2, Figure 3, and Figure 7). We propose that lowering SPT activity increases CLS, at least in part, by reducing the concentration of LCBs thereby lowering the activity of Pkh1/2 and Sch9 which programs cells for an extended lifespan (Figure 1A). This scenario is supported by data showing that myriocin treatment or down-regulation of *tetO7-LCB1* gene expression diminishes Pkh1/2-dependent phosphorylation of Sch9 residue T570, essential for activity (Figure 5) and reduces phosphorylation of the Sch9 substrate Dot6 (Figure 5I). In addition, as a surrogate for Sch9 activity in vivo, we measured processes that play key roles in lifespan and are regulated by Sch9. All processes including resistance to heat, oxidative and acetic acid stress (Figure 2, Figure 3, Figure 6A and 6B), cell size (Figure S2 in Text S1), mitochondrial oxygen consumption (Figure 6C), and genome stability (Figure 6D and 6E) responded to down-regulation of sphingolipid synthesis in a manner indicative of reduced Sch9 activity.

Deletion of *PKH2* was recently shown to extend CLS [34]. We verified this finding (Figure 5A) and show that phosphorylation of Sch9 T570 is reduced in *pkh2Δ* cells (Figure 5B). Finally, we show that cells with low Pkh kinase activity are much more sensitive to growth inhibition by myriocin or Dox than are cells with higher Pkh activity (Figure 5D and Figure S4 in Text S1). Together these data add further support for a signaling pathway comprised of sphingolipids→Pkh1/2→Sch9→CLS.

While most of our data suggest that down-regulating sphingolipid synthesis increases lifespan by Sch9-dependent mechanisms, the finding that myriocin causes a mobility shift in Sch9, indicative of reduced phosphorylation, and increases the CLS of *sch9Δ* cells argues for some aspects of CLS extension operating by Sch9-independent mechanisms. Down-regulation of the protein kinase A [45] pathway is the most likely way for sphingolipids to affect lifespan independently of Sch9, since these two pathways have been known to interact ever since the initial discovery of Sch9 [46]. Also, the pathways are known to interact in a way that extends CLS, since *ras2Δ sch9Δ* double mutant cells have a longer lifespan than the single mutants [30]. This interaction phenotype is similar to what we find with myriocin treatment of *sch9Δ* cells (Figure 7A). Recent publications have detected biochemical links between the Pkh1/2-Sch9 and PKA pathways [24], [25], [47] and these may explain how myriocin increases the lifespan of *sch9Δ* cells, but further effort is needed to determine if this is actually the case.

In addition to LCBs, complex sphingolipids (IPCs and perhaps MIPCs) may be regulating CLS, since their concentration is lowered along with LCBs when SPT activity is down-regulated (Figure 4C, 4D, and 4E). A multi-membrane spanning protein Nce102 has been proposed to sense complex sphingolipids and negatively regulate Pkh1/2 [26]. According to this proposal, a reduction in complex sphingolipids should increase Pkh1/2 activity by releasing Nce102-mediated down-regulation. As a consequence, Sch9 activity should increase; however, this would not enhance CLS and is contrary to our finding that reducing sphingolipids decreases Sch9 activity. Thus, Nce102 is unlikely to be mediating the increase in CLS that we observe.

Another group has also questioned the role of LCBs in activating Pkh1/2 kinase activity [27]. These authors found that short-term, acute dose myriocin treatment of cells failed to reduce Pkh1/2-mediated phosphorylation of residue T504 in the Ypk1 kinase. And recently, short-term, acute dose treatment of cells with myriocin did not decrease Pkh1/2-mediated phosphorylation of T570 in Sch9 [25]. In contrast to these results, we show that lowering the rate of sphingolipid synthesis by long-term, low dose myriocin treatment or down-regulation of *LCB1* expression reduces Pkh1/2-mediated phosphorylation of T570 (Figure 5) and, in addition, myriocin treatment reduces phosphorylation of the Sch9 substrate Dot6 (Figure 5I). These results clearly support the idea that sphingolipids regulate lifespan by reducing Pkh1/2-mediated phosphorylation of T570 thereby reducing Sch9 activity. Further work is required to determine which sphingolipids control Sch9 activity and CLS (Figure 1A).

Studies of the *ISC1* gene also implicate sphingolipids in controlling lifespan. Isc1 hydrolyzes the polar head group from complex sphingolipids to yield ceramides. The CLS of *isc1Δ* cells is reduced partly because of mitochondrial dysfunctions [48], but CLS can be extended in these cells even beyond the wild-type value by deleting the *SIT4* gene. Sit4 is the catalytic subunit of a PP2A-type protein phosphatase whose activity is down-regulated by TORC1 but up-regulated by ceramides (Figure 1A). In our experiments, enhancement of CLS by down-regulating sphingolipid synthesis in both wild-type and *sch9Δ* cells may involve Sit4, because reducing the abundance of IPCs, and perhaps MIPCs (Figure 4), may lower the amount of ceramide generated by Isc1. This should in turn lower Sit4 activity and increase CLS just like deletion of *SIT4* (Figure 1A). Finally, other studies implicate complex sphingolipids in CLS, since *ipt1Δ* and *skn1Δ* cells, which lack the most complex sphingolipid M(IP)_2_C, have a slight increase in CLS and deletion of both genes yields an additive increase in CLS [49]. It remains to be determined how a reduction in M(IP)_2_C increases CLS, but this effect is probably real as *skn1Δ* cells ranked high in a survey of lifespan in the yeast single-deletion strain set [1].

Sphingolipids have been implicated also in RLS. The first *S. cerevisiae* gene found to regulate RLS, *LAG1* (Longevity-Assurance Gene 1) [50], was later shown to encode a component of ceramide synthase which catalyzes ceramide synthesis from LCBs and fatty acids (Figure S1 in Text S1) [51], [52]. Deleting *LAG1* increases RLS but has no effect on CLS [1]. Since the mechanism for increased RLS without increased CLS is unknown, it is difficult to relate these data to our CLS data.

In cases where a decrease in LCBs has perturbed a cellular process in yeast it has been possible to restore the process by adding LCBs to the culture medium [53]–[55]. This strategy suggests that exogenous PHS should prevent the increase in CLS produced by Dox treatment of *tetO7-LCB1* cells. In actuality, low levels of PHS (10–500 nM) did not thwart the increase in CLS and higher doses (10 µM) actually enhanced CLS. Exogenous PHS is likely to enhance CLS by inducing the general stress response via activation of genes with stress response elements (STREs) in their promoter, as previously reported [53], [56]. In addition, exogenous PHS is known to down-regulate amino acid and other nutrient transporters in yeast [57], [58] and this is likely to increase lifespan since amino acid or glucose deprivation enhance yeast lifespan [1], [59].

### Lifespan regulation by myriocin and CR

Because CR is the standard for judging interventions that extend lifespan, we determined if myriocin enhances lifespan in CR-treated cells. We found that myriocin treatment does enhance CLS in cells grown under moderate (0.5% glucose, Figure S3 in Text S1) or extreme CR (water) conditions (Figure 7B). These results are similar to the recent finding that rapamycin treatment increases the lifespan of caloric restricted *Drosophila* [3] and they have implications for human health. For example, it may be possible to reduce disease incidence and increase human lifespan by combining a drug with low level CR or with a CR mimetic such as rapamycin. Furthermore, a combination regime may reduce disease incidence in genotypic backgrounds which are unresponsive to single treatment regimes.

### Sphingolipids and lifespan in multicellular eukaryotes

Increasing lifespan by using a pharmacological agent to down-regulate sphingolipid synthesis may be applicable to other organisms in addition to yeasts much as rapamycin treatment has been used to down-regulate the TOR pathway and increase lifespan in yeast, worms, flies and mice [1]–[3], [10], [18]. The sphingolipids that regulate lifespan in yeast may not be present in mammals and, thus, lifespan extension may require modulating another type of mammalian sphingolipid [11]. Ceramides and sphingosine-1-phosphate are the most widely appreciated sphingolipid signaling molecules in mammals [11], [12]. Ceramides are thought to promote age-related diseases since their concentration increases with age in many mammalian cells and tissues where they regulate processes that may control lifespan [60]–[64]. Recently long-chain hexosylceramides and lactosylceramides have been implicate in aging and lifespan in mice and cultured human cells [65]. The challenge will be to determine if mammalian lifespan can be extended by preventing the increase in ceramides or whether up or down-modulating the concentration of other sphingolipids will extend lifespan. There is, however, precedent for extending lifespan in multicellular eukaryotes through modulation of sphingolipids: inactivation of the Dacer gene, which encodes an alkaline ceramidase, has been shown to increase lifespan in *Drosophila* [66].

As mutations and chromosomal rearrangements promote mammalian tumor cell formation, our demonstration that myriocin treatment reduces the rate of mutation and gross chromosomal rearrangements in yeast (Figure 5D and 5E) could lead to novel preventive measures and treatments for human cancers and thereby enhance lifespan.

## Materials and Methods

### Yeast strains, media, and plasmids

Yeast strains used in our studies are listed in Table S1 in Text S1. Viability assays were done by using YPD agar plates (2% yeast extract, 1% peptone, 2% dextrose and 2% agar). For CLS and stress resistance assays, cells were grown in SDC medium supplemented with a 4-fold excess of the tryptophan, leucine, uracil and histidine to avoid possible artifacts due to auxotrophic deficiencies of the strain [30]. For initial experiments, SDC medium was lightly buffered to pH 6.0 (1.6 gm NaH_2_PO_4_ • H_2_O/liter), while in later experiments it was more strongly buffered with succinic acid (200 mM, final pH 4.5). Initially the concentration of iron in SDC medium was 1.23 µM (1× yeast nitrogen base) but in later experiments the concentration was raised 3-fold to avoid iron-starvation and reduced viability. In summary, SDC medium, pH 6.0, 1× iron, was used for the experiments shown in Figure 1, Figure 2, and Figure 3A–3F, while the same medium, but with 3× iron, was used for the experiments shown in Figure 3H, Figure 4, and Figure 6A. For the experiments shown in Figure 3G, 3H, Figure 5, Figure 6C–6E, Figure 7, and Figures S2, S3, S4 in Text S1 the medium was SDC, pH 4.5, 3× iron.

The pRS315 and pRS316 vectors [67] were used to make pRS315ADE2 by ligating a 2.3 Kb *PstI-SpeI* fragment carrying *ADE2* into the corresponding sites of pRS315. Similarly, a genomic *EcoRI-XhoI* fragment was ligated to pRS316 cut with the same enzymes to make pRS316-PKH1 and a genomic *BamHI-SalI* fragment was ligated to pRS316 cut with the same enzymes to make pRS316-PKH2. pLCB1-5 was made by ligating a 4 Kb *NruI-SacI* chromosomal fragment containing *LCB1* and its promoter [68] to pRS315ADE2 digested with *SacI* and *NaeI* (made blunt ended). pRS315-LCB2-B7 was made by ligating a 7 Kb *BamHI* chromosomal fragment containing *LCB2* and its promoter [69] to pRS315 digested with *BamHI*. The plasmid encoding HA-tagged Dot6 has been described [36].

### Lifespan assays

CLS was measured as previously described [30]. Drug treatments were performed in medium containing a final concentration of 0.3% ethanol (ethanol plus drug). This was done be first adding a calculated volume of 95% ethanol to the medium followed by diluting a stock solution of myriocin (200 µg/ml in 95% ethanol, Sigma, # M1177) or doxycycline (100 µg/ml in 50% ethanol, Sigma, # D9891) to yield the desired concentration of drug (indicated in figure legends). Drugs were stored at −20°C and warmed to room temperature before dilution into medium, followed by thorough mixing. Cells grown overnight at 30°C in SDC medium were diluted into 25 ml of medium (125 ml flask) to give an initial A_600 nm_ of 0.002 or as indicated in figure legends. Cultures were incubated at 30°C in an air bath shaker (220 rpm) for three days and cell viability was measured (CLS day 1) by diluting and spreading cells on YPD plates. After 2–3 days of incubation at 30°C colonies were counted and expressed a fraction of the day 1 value. Statistical significance was determined by using the two-tailed Student's *t*-test.

### Stress resistance and oxygen consumption assays

Cells grown in SDC medium for the time indicated in figure legends were diluted to an A_600 nm_ of 1.0 in K-phosphate buffer (pH 6.0), treated with hydrogen peroxide for 60 min at room temperature with mixing, serially diluted (10-fold), spotted onto YPD plates, and incubated at 30°C for 2–3 days. For thermal stress resistance, cells were serially diluted (10-fold dilution starting with an A_600 nm_ = 1), spotted onto YPD plates, and incubated at either 55°C (heat-shocked) or 30°C (control) for 45–180 min. Plates were incubated for 2–3 days at 30°C. For acetic acid resistance, cells from day 1 (72 hrs of incubation) of a CLS assay were treated with acetic acid as specified in the legend to Figure 6 for 2 hours at 30°C, followed by dilution and plating on YPD plates to measure colony forming units (CFUs). CFUs of the treated samples were expressed as a percentage of the untreated samples.

Oxygen consumption was measured on cells grown from an A_600 nm_ of 0.005 to 1 at which time the cell concentration was quantified by counting cells in a Petroff-Hausser counting chamber, cell size was measured as described in Supplemental Information and oxygen consumption was measured by using an Oxytherm System [38]. Data are expressed as nmol of oxygen consumed per min per total volume of 10^6^ DBY746 cells.

### Age-dependent DNA damage measurements

The frequency of spontaneous canavanine-resistant mutants (Can^r^) mutants was measured by using a published assay [42]. Cells were grown as described for CLS experiments and assayed for CFUs except that cultures were started at a density of 0.05 A_600 nm_ units/ml and the SDC medium was buffered with succinate (200 mM, pH 4.5) and contained 3.69 µM iron (3× normal). These changes were necessary to prevent parental cells from dying so rapidly that they behaved like *sgs1* mutant cells. Mutant *sgs1* cells were grown in medium containing 300 ng/ml of myriocin. The frequency of Can^r^ mutants was measured on the days indicated in Figure 6D. To identify Can^r^ mutants, a sample of the culture was centrifuged and the cell pellet was washed once with sterile water and plated on selection medium (SDC-ARG supplemented with 60 µg/ml L-canavanine sulfate)s. After 3–4 days of incubation at 30°C, the colonies were counted and expressed as the ratio to total viable cells. GCRs were assayed as described previously [42]. Strains carried a *URA3* cassette at the *HXT13* locus which is located 7.5 kb from the *CAN1* gene towards the telomeric of chromosome V. The experimental protocol was similarly to the one for measuring Can^r^ mutations but the frequency of simultaneous loss of *CAN1* and *URA3* was measured by plating cells on SDC plates containing 1 mg/ml 5-fluoroorotic acid (5FOA) and 60 µg/ml L-canavanine sulfate [70].

### Analysis of LCBs, LCBPs, and complex sphingolipids

To extract LCBs and LCBPs, cells were grown as described for CLS assays and at the indicated time points 10 A_600 nm_ units of cells were treated by slowly adding cold TCA to a final concentration of 6%. After incubating on ice for 5 min, cells were collected by centrifugation, washed twice with cold water, and suspended in 500 µl of fresh 80 mM triethylamine (made in HPLC grade absolute ethanol). Samples were sonicated in a water bath for 5 min at 37°C, heated for 30 min at 65°C, centrifuged and the supernatant fluid (150 µl) was transferred to an HPLC sample vial insert. AQC (6-aminoquinolyl-N-hydroxysuccinimidyl carbamate) reagent (30 µl) was added and samples were treated at room temperature for 40 min followed by addition of 20 µl of 1.5 N KOH (made in MeOH) and incubation at 37°C for 30 min. The pH was adjusted by adding 20 µl of 1.74 M acetic acid (made in MeOH) and 40 µl of the sample were assayed by HPLC [71].

Complex sphingolipids were analyzed as described previously [72] except for three changes. First, cells were grown in SDC medium (pH 6, 3.69 µM Fe) from 0.005 to 1 A_600 nm_ units to produce uniform radiolabeling. Second, the concentration of [2-^3^H]myoinositol (American Radiochemicals, Inc., catalog #ART116) in the growth medium was 40 µCi/ml. Finally, each lane on the thin-layer plate contained 20 µL of non-radioactive yeast sphingolipids (0.5 mg/ml), which after charring, were used to verify the location of radioactive sphingolipids.

### Protein extraction and Western blotting analysis

Cells were grown as described for a CLS assay and cell-free yeast extracts were prepared by using a modified published procedure [73]. Ten A_600 nm_ units of cells were treated with cold TCA as described above, suspended in 400 µl of water followed by the addition of 400 µl of 0.2 M NaOH. Thoroughly mixed samples were incubated 5 min at room temperature, concentrated by centrifugation and suspended in 200 µl of lysis buffer (0.06 M Tris-HCl, pH 6.8, 5% glycerol, 2% SDS, 1% β-mercaptoethanol). After heating at 95°C for 5 min, samples were centrifuged and the protein concentration of the supernatant fluid was determined by using the DC protein assay kit (Bio-Rad Laboratories). Samples (50 µg of protein) electrophoresed on a 9% SDS-PAGE gel were transferred for 1.5 hours onto a PVDF membrane (Millipore Immobilon) by using a Bio-Rad semi-dry transfer system. For the blots shown in Figure 5I and 5J the acrylamide-bisacrylamide ratio was changed from the normal 37.5∶1 ratio to 172∶1. Membrane blocking and antibody binding were done in TBST (20 mM Tris, 150 mM NaCl, 0.1% Tween-20, pH 7.5) containing 5% nonfat dry milk. Membranes were incubated for 2 hrs at room temperature with custom made polyclonal rabbit anti-Sch9 antibodies (Batch 2872, 1∶1000, OpenBiosystems), phosphor-specific anti-Sch9T570 antibodies (1∶10,000 dilution) [20], anti-HA antibodies (clone 3F10, 1∶5000, Roche) or commercial monoclonal mouse anti-Vma2 (1∶3,000 dilution) or anti-Vph1 (1∶1000 dilution) antibodies (Molecular Probes). Other antibodies included alkaline phosphatase-linked anti-rabbit or anti-mouse IgG (1∶5000, Sigma-Aldrich). Fluorescent signals from membranes exposed to an ECF substrate (Amersham Biosciences) were analyzed by using a PhosphorImager (Amersham Biosciences) and quantified by using ImageQuaNT Software.

## Supporting Information

Text S1 Supporting information, including supporting figures, table, and references. Figure S1: Outline of sphingolipid metabolism in *Saccharomyces cerevisiae*. Metabolic intermediates and complex sphingolipids are shown in bold font, genes are shown in italics and enzyme names are in regular lettering. Structures of compounds have been presented previously [74], [75]. Figure S2: Myriocin treatment decreases cell size. DBY746 cells were grown with and without myriocin (Myr) as in a CLS assay using SDC medium (pH 4.5, 3X iron). After 72 hrs of incubation, cells were stained directly with Calcofluor white M2R (25 µ/ml) and photographed at room temperature by using a Nikon Eclipse E600 fluorescence microscope equipped with a Plan Apo 100× 1.40 oil immersion objective, a SPOT RT 9.0 Monochrome-6 camera and SPOT basic software. For measurements, we excluded extremely large or small cells and cell diameter was calculated by measuring and averaging the long and short axes (perpendicular to each other) of each cell as described previously [30]. The median diameter for one hundred cells is indicated by a horizontal bar in the scatter plot. Figure S3: CLS of WT (DBY746) cells grown in SDC medium (pH 4.5, 3X iron) with CR (0.5% glucose) or without CR (NR, 2% glucose) +/− myriocin (Myr) treatment. Data represent the mean ± SEM of survival (* p<0.05, ** p<0.01, No Myr vs 450 or 600 ng/ml Myr, CR cultures). Figure S4: Sphingolipids activate the Pkh1/2 protein kinases. (A) Growth sensitivity was measured by diluting cells from CLS day 1 (10-fold serial dilution from left to right), spotting onto YPD plates containing the indicated concentration of myriocin, and incubating 3 days at 30 °C. Strains are: WT (R1158, LCB1), tetO7-LCB1 (RCD956), tetO7-LCB1/pkh1Δ (RCD1048), and tetO7-LCB1/pkh2Δ (RCD1051). (B) Same strains as used in A, but spotted onto YPD plates containing Dox. Table S1: Strains used in this study.
(DOCX)
