# Supplementary material for: Down-Regulating Sphingolipid Synthesis Increases Yeast Lifespan
Source: PLoS Genet. 2012 Feb 2;8(2):e1002493. doi: 10.1371/journal.pgen.1002493 (PMC3271065; doi:10.1371/journal.pgen.1002493)
Supplement: Text S1 — Supporting information, including supporting figures, table, and references. Figure S1: Outline of sphingolipid metabolism in Saccharomyces cerevisiae. Metabolic intermediates and complex sphingolipids are shown in bold font, genes are shown in italics and enzyme names are in regular lettering. Structures of compounds have been presented previously [74], [75]. Figure S2: Myriocin treatment decreases cell size. DBY746 cells were grown with and without myriocin (Myr) as in a CLS assay using SDC medium (pH 4.5, 3X iron). After 72 hrs of incubation, cells were stained directly with Calcofluor white M2R (25 µ/ml) and photographed at room temperature by using a Nikon Eclipse E600 fluorescence microscope equipped with a Plan Apo 100× 1.40 oil immersion objective, a SPOT RT 9.0 Monochrome-6 camera and SPOT basic software. For measurements, we excluded extremely large or small cells and cell diameter was calculated by measuring and averaging the long and short axes (perpendicular to each other) of each cell as described previously [30]. The median diameter for one hundred cells is indicated by a horizontal bar in the scatter plot. Figure S3: CLS of WT (DBY746) cells grown in SDC medium (pH 4.5, 3X iron) with CR (0.5% glucose) or without CR (NR, 2% glucose) +/− myriocin (Myr) treatment. Data represent the mean ± SEM of survival (* p<0.05, ** p<0.01, No Myr vs 450 or 600 ng/ml Myr, CR cultures). Figure S4: Sphingolipids activate the Pkh1/2 protein kinases. (A) Growth sensitivity was measured by diluting cells from CLS day 1 (10-fold serial dilution from left to right), spotting onto YPD plates containing the indicated concentration of myriocin, and incubating 3 days at 30 °C. Strains are: WT (R1158, LCB1), tetO7-LCB1 (RCD956), tetO7-LCB1/pkh1Δ (RCD1048), and tetO7-LCB1/pkh2Δ (RCD1051). (B) Same strains as used in A, but spotted onto YPD plates containing Dox. Table S1: Strains used in this study. (DOCX) [file pgen.1002493.s001.docx]

**Text S1**

**Down-regulating Sphingolipid Synthesis Increases Yeast Lifespan**

**Xinhe Huang, Jun Liu and Robert C. Dickson**


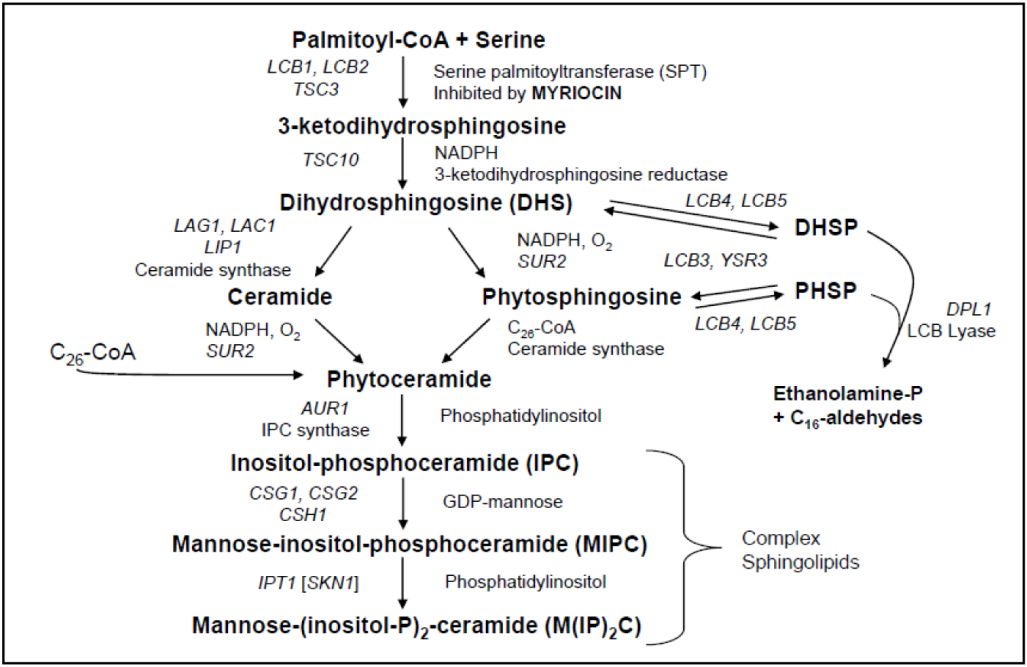


**Figure S1.**


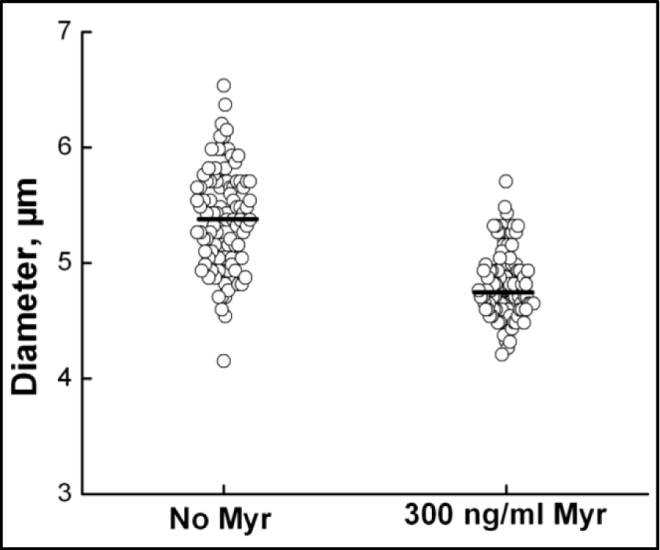


**Figure S2.**


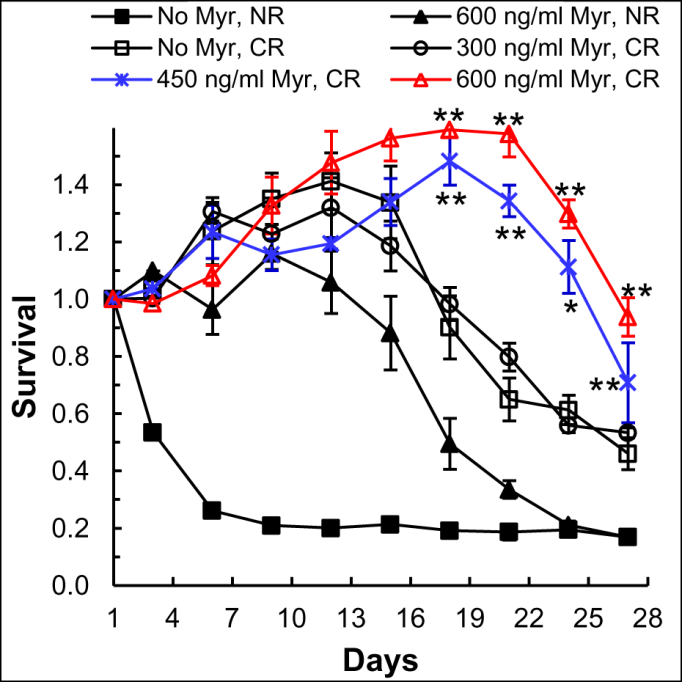


**Figure S3.**


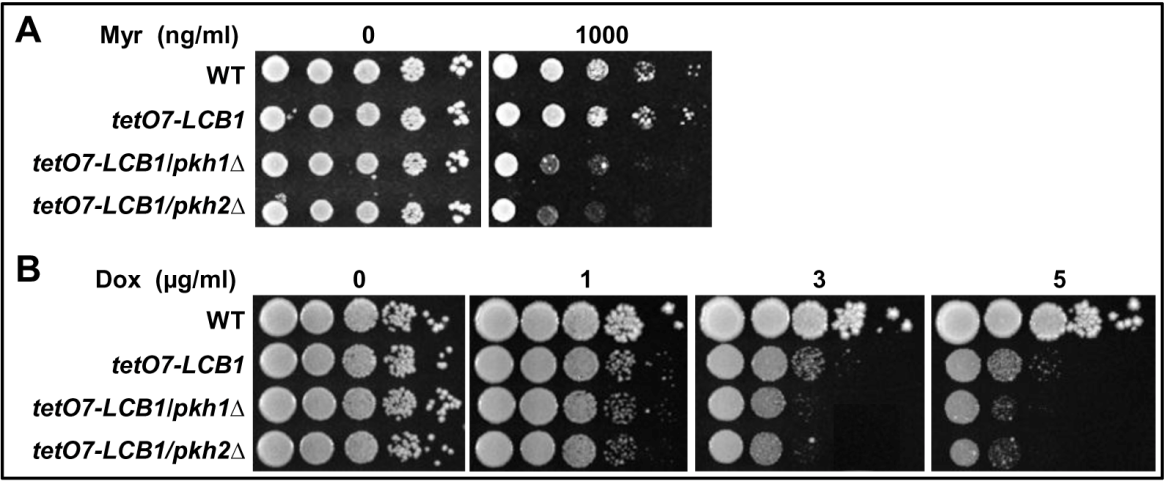


**Figure S4**.

**Table S1.** Strains used in this study.

| **Strains** | **Genotype** | **Source** |
| --- | --- | --- |
| DBY746 | *MATα leu2-3,112 his3Δ1 trp1-289 ura3-52 GAL^+^* | [4] |
| PF102 | DBY746 with *sch9∷URA3* | “ |
| BY4741 | *MATa his3Δ1 leu2Δ0 ura3Δ0 met15Δ0* | [4,5] |
| R1158 | BY4741 with *URA3∷CMV-Tta* | [6] |
| RCD952 | R1158 with *KAN∷tetO7∷SCH9* | This study |
| RCD956 | R1158 with *KAN∷tetO7∷LCB1* | “ |
| RCD957 | R1158 with *KAN∷tetO7∷LCB2* | “ |
| RCD994 | R1158 with *KAN∷tetO7∷RIM15* | “ |
| RCD1009 | DBY746 with *hxt13∷URA3* | [7] |
| RCD1010 | DBY746 with *hxt13∷URA3, sgs1∷HIS3* | “ |
| RCD1011 | DBY746 with *hxt13∷URA3 sgs1∷HIS3 sch9∷TRP1* | “ |
| RCD1012 | DBY746 with *hxt13∷URA3, sch9∷TRP1* | “ |
| RCD1052 | RCD956 with *ura3::HIS3::CMV-Tta* | This study |
| RCD1048 | RCD956 with *pkh1::NAT* | “ |
| RCD1051 | RCD956 with *pkh2::NAT* | “ |
| 15Dau | *MATa ura3 leu2 trp1 his2 ade1* | [8] |
| INA106-3B | *MATa ura3 leu2 trp1 his2 ade1 pkh1^D398G^ pkh2::LEU2* | “ |
